# A High-Fidelity mmWave Radar Dataset for Privacy-Sensitive Human Pose Estimation

**DOI:** 10.3390/bioengineering12080891

**Published:** 2025-08-21

**Authors:** Yuanzhi Su, Huiying (Cynthia) Hou, Haifeng Lan, Christina Zong-Hao Ma

**Affiliations:** 1 Department of Building Environment and Energy Engineering, The Hong Kong Polytechnic University, Hong Kong SAR, China; yuanzhi.su@connect.polyu.hk (Y.S.); haifeng.lan@connect.polyu.hk (H.L.); 2 Department of Biomedical Engineering, The Hong Kong Polytechnic University, Hong Kong SAR, China; czh.ma@polyu.edu.hk; 3 Research Institute for Sports Science and Technology, The Hong Kong Polytechnic University, Hong Kong SAR, China; 4 Research Institute for Smart Ageing, The Hong Kong Polytechnic University, Hong Kong SAR, China

**Keywords:** mmWave radar, privacy-preserving sensing, human pose estimation

## Abstract

Human pose estimation (HPE) in privacy-sensitive environments such as healthcare facilities and smart homes demands non-visual sensing solutions. Millimeter-wave (mmWave) radar emerges as a promising alternative, yet its development is hindered by the scarcity of high-fidelity datasets with accurate annotations. This paper introduces mmFree-Pose, the first dedicated mmWave radar dataset specifically designed for privacy-preserving HPE. Collected through a novel visual-free framework that synchronizes mmWave radar with VDSuit-Full motion-capture sensors, our dataset covers 10+ actions, from basic gestures to complex falls. Each sample provides (i) raw 3D point clouds with Doppler velocity and intensity, (ii) precise 23-joint skeletal annotations, and (iii) full-body motion sequences in privacy-critical scenarios. Crucially, all data is captured without the use of visual sensors, ensuring fundamental privacy protection by design. Unlike conventional approaches that rely on RGB or depth cameras, our framework eliminates the risk of visual data leakage while maintaining high annotation fidelity. The dataset also incorporates scenarios involving occlusions, different viewing angles, and multiple subject variations to enhance generalization in real-world applications. By providing a high-quality and privacy-compliant dataset, mmFree-Pose bridges the gap between RF sensing and home monitoring applications, where safeguarding personal identity and behavior remains a critical concern.

## 1. Introduction

Human pose estimation (HPE) has emerged as a foundational technology enabling transformative applications across healthcare monitoring [1,2,3], human–computer interaction [4,5,6], and smart environment systems [7,8,9]. In clinical contexts, continuous pose tracking facilitates early detection of critical events such as falls in elderly populations [10,11], while in residential settings, it supports intuitive gesture-based control of domestic appliances [5,12]. However, the predominant reliance on visual sensing modalities—particularly RGB cameras [13,14]—introduces significant limitations in privacy-sensitive environments. These systems inherently capture personally identifiable information, raising serious ethical and regulatory concerns in contexts like patient rooms, bathrooms, or private residences, where confidentiality is paramount. Moreover, their performance degrades substantially under suboptimal lighting conditions, limiting reliability in nocturnal care scenarios.

Millimeter-wave (mmWave) radar technology [15,16] presents a promising alternative, operating through radio frequency signals rather than optical capture. This modality offers two distinct advantages: immunity to lighting variations and inherent privacy preservation through non-visual data acquisition. By generating sparse 3D point clouds representing spatial coordinates without capturing biometric identifiers, mmWave systems fundamentally avoid the privacy violations associated with visual sensors. Despite these benefits, advancement in mmWave-based HPE has been constrained by the absence of high-fidelity datasets that simultaneously satisfy two critical requirements: precise annotations and coverage of wide daily movements.

Existing datasets exhibit notable limitations in this domain. The HuPR benchmark [17] leverages synchronized RGB cameras to generate pseudo-labels via pre-trained vision models, inheriting annotation inaccuracies while compromising privacy through visual data capture. Similarly, the dataset introduced by Sengupta et al. [18] relies on Microsoft Kinect for ground truth, introducing depth-sensing errors estimated at >50 mm positional deviation. More fundamentally, current resources lack comprehensive coverage of medically critical scenarios such as falls—events that demand high detection reliability but occur predominantly in privacy-sensitive contexts. This gap between technological potential and available training resources has impeded the development of robust mmWave HPE systems for real-world applications where privacy and accuracy are significant.

**Figure 1:**
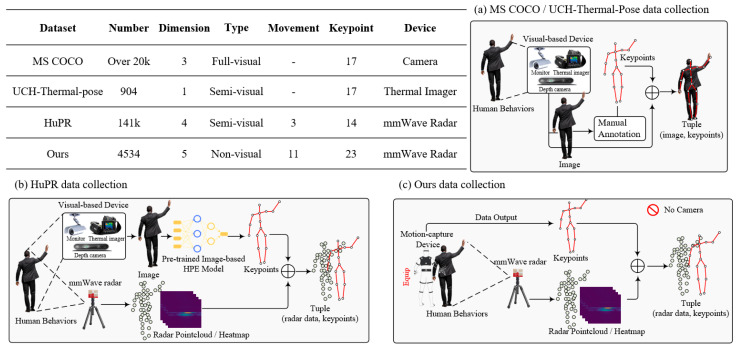
Comparative framework of human pose dataset acquisition: visual vs. non-visual paradigms. (**a**) Traditional visual/semi-visual methods (MS COCO and UCH-Thermal-Pose). (**b**) Semi-visual pseudo-labeling (HuPR). (**c**) Proposed privacy-centric framework with visual-free annotation.

To address these challenges, we present mmFree-Pose, a novel dataset specifically designed for privacy-sensitive human pose estimation using mmWave radar. The specific comparison is shown in Figure 1. Our acquisition framework employs synchronized mmWave radar and inertial motion-capture sensors, eliminating optical sensors entirely from the data collection pipeline. This approach ensures fundamental privacy protection while achieving medical-grade annotation precision. The dataset comprises 4534 temporally aligned samples capturing 10+ actions performed by three subjects, encompassing both basic movements (e.g., arm waving and leg lifts) and complex scenarios (e.g., forward/backward falls) in multiple orientations relative to the radar. Each sample provides five-dimensional point cloud data (x, y, z, velocity, and intensity) paired with precise 23-joint skeletal annotations.

The primary contributions of this work are threefold:

- We establish a privacy-by-design data acquisition paradigm, enabling high-fidelity pose estimation in privacy-sensitive environment.
- We establish the first mmWave radar dataset with high-fidelity full-body pose annotations generated without any optical or visual data.
- Experimental validation demonstrates executive performance on complex poses, with testing on several benchmark models, achieving a significant performance.

Collectively, this resource bridges a critical gap in sensing infrastructure for applications where privacy constraints preclude visual monitoring, such as healthcare facilities or smart homes.

The remainder of this paper is structured as follows: Section 2 examines related work on privacy-preserving human pose estimation, mmWave-based pose estimation, and identifies specific limitations in existing datasets and inertial motion capture for annotation. Section 3 details the sensor synchronization methodology and data collection protocol. Section 4 presents benchmark evaluations and comparative analysis. Section 5 discusses practical applications, limitations, and future research directions. Concluding remarks are provided in Section 6.

## 2. Related Work

### 2.1. Privacy-Preserving Human Pose Estimation

Traditional vision-based human pose estimation (HPE) [4,6,19,20,21,22,23,24,25,26,27] methodologies face fundamental limitations in privacy-sensitive contexts. RGB-D systems [26] like Microsoft Kinect capture detailed body silhouettes, risking biometric identifier exposure that violates relevant regulations in healthcare settings. Infrared alternatives [28,29] offer partial mitigation by eliminating texture and color information, but thermal imagery still retains spatial heat distributions that, when combined with shape priors, can be reverse-engineered to approximate human identity or body type. Recent RF-based approaches [30,31] using Wi-Fi or ultrawideband radar provide anonymity through signal-scattering patterns, yet suffer from low spatial resolution (>20 cm error), making them unsuitable for fine-grained tasks, particularly in applications requiring higher localization accuracy. In this landscape, millimeter-wave (mmWave) radar emerges as a balanced solution, offering centimeter-level spatial resolution through short-wavelength signal propagation, while preserving subject anonymity by avoiding any visual or thermal representation. Its non-invasive nature aligns well with privacy-by-design frameworks, making it particularly suitable for sensitive environments like bathrooms, bedrooms, or eldercare facilities. Yet paradoxically, many existing mmWave HPE implementations still rely on optical sensors (RGB cameras and depth sensors) [17,18,32,33] to generate annotated ground truth, introducing privacy compromise at the training phase even if inference is performed using radar alone. This reliance not only undermines privacy claims but also restricts the deployment of such models in privacy-critical applications due to regulatory scrutiny.

Our work directly addresses this conflict by proposing a fully visual-free data acquisition pipeline, thereby eliminating the need for camera-based annotation while maintaining high fidelity via inertial motion capture. This resolves the long-standing trade-off between privacy preservation and annotation accuracy, a crucial step toward enabling ethically compliant, real-world human monitoring solutions.

### 2.2. Fundamentals of mmWave Radar Sensing

Millimeter-wave (mmWave) radar [15,16] operates by emitting high-frequency radio waves (typically 60–80 GHz) and analyzing reflections from objects [20]. Unlike cameras, it captures motion through radio signals rather than light. The fundamental operating principle of mmWave radar involves transmitting electromagnetic waves in the millimeter-wave range and analyzing the signals reflected from objects within the radar’s field of view. The radar system transmits a millimeter-wave signal through an antenna array. This signal is modulated to increase linearly over a defined time interval as it propagates through the environment and interacts with objects in its path. Depending on the material properties and surface geometry of these objects, a portion of the signal is reflected back toward the radar. The receiving antenna captures these reflected signals and combines them with a segment of the transmitted signal, producing an intermediate frequency (IF) signal.

These IF signals are digitized and processed through a series of Fast Fourier Transforms (FFTs) to resolve range, Doppler velocity, and angle information. Specifically, a 3D radar point is formed using range FFT (distance resolution), Doppler FFT (velocity measurement), and angle FFT (azimuth/elevation angles). Then Constant False Alarm Rate (CFAR) detection isolates valid human targets from noise, followed by coordinate conversion to Cartesian space. Hence, each point is represented as p={x,y,z,velocity,intensity}, where the coordinates $\left(x,y,z\right)$ define the object’s location, and the additional attributes indicate the object’s velocity and signal intensity within the radar’s field of view. By repeating this process, a series of signals received by mm-wave radar can be converted into a point set, which can be represented as point cloud $P=\{p_{i}\left|p_{i}=\{x_{i},y_{i},z_{i},{\mathrm{velocity}}_{i},{\mathrm{intensity}}_{i}\}\in R^{5},i=1,2,\ldots ,N\right\}$, where N indicates the number of points.

For generating 3D point clouds where each point represents a detected surface location, velocity, and reflectivity, this technology leverages two key physical properties:

(1) High-frequency waves provide fine spatial resolution (centimeter-level), enabling detection of small body movements like joint rotations.
(2) Signal time-of-flight measurements calculate object distance, while Doppler shifts in reflected waves reveal limb velocity.

For human pose estimation, mmWave radar offers inherent privacy advantages: its output contains only geometric coordinates without visual details (e.g., facial features or skin texture), making it compliant for sensitive settings. Additionally, it functions reliably in darkness and through light obstructions (e.g., clothing or curtains), addressing critical limitations of optical sensors. While challenges like sparse data points remain, these characteristics position mmWave as a unique enabler for ethical human monitoring where cameras cannot be deployed.

### 2.3. mmWave Radar Datasets for HPE

Existing mmWave HPE datasets exhibit critical limitations in privacy compliance and annotation quality. The emergence of mmWave radar technology has opened new avenues for HPE and provides a robust alternative to visual sensors. At present, several datasets have been developed to explore this potential. For instance, Sengupta et al. [18] proposed an HPE dataset using mmWave radar, which consists of radar reflection data encoded as RGB images, with pixel values representing 3D coordinates and reflection intensity, along with ground-truth skeletal joint data captured via Microsoft Kinect. Xue et al. provided a detailed dataset for 3D mesh reconstructions from radar data, offering a valuable resource for developing and benchmarking 3D HPE algorithms. Another notable dataset, HuPR [17], incorporates the synchronized vision and radar components for cross-modality training. HuPR leverages pre-trained 2D pose estimation networks on RGB images to generate pseudo-label annotations for radar data, thereby avoiding manual labeling efforts and enhancing training efficiency.

Despite these valuable contributions, existing radar datasets suffer from several drawbacks, such as limited diversity in human poses and inaccurate data annotations, all of which hinder broader experimentation and development in the field. In contrast, this paper presents a comprehensive dataset that includes a wide range of human poses, from simple to complex, and features high-fidelity annotations obtained via motion-capture technology. This approach ensures accurate ground-truth data, free from the limitations of model-generated annotations, and provides a richer, more diverse dataset for training robust mmWave radar-based HPE models. By directly addressing the shortcomings of existing datasets, our work advances the capabilities of RF-based HPE technologies, paving the way for real-world applications.

### 2.4. Inertial Motion Capture for Annotation

Inertial measurement unit (IMU)-based systems [34] offer privacy-compliant alternatives for motion capture. The VDSuit-Full system deployed in this work utilizes 23 wireless IMUs to reconstruct skeletal poses without optical inputs. Comparable systems like Xsens MVN [35] provide medical-grade kinematics but require proprietary software that impedes annotation extraction. Recent work [36,37,38] demonstrates IMU-based operating-room monitoring, validating inertial systems’ clinical reliability. However, IMUs are susceptible to integration drift, quadrature errors, and cross-axis sensitivity over time, particularly during dynamic sequences. Existing works have attempted to model and mitigate such effects via compensation schemes [39].

Despite limitations such as potential drift over long sequences and reliance on initial calibration, IMUs remain particularly well-suited for annotating mmWave radar data. Their high temporal resolution compensates for radar’s lower frame rate and point cloud sparsity, while their numerical output avoids any form of biometric reconstruction. This synergy allows for accurate spatial–temporal alignment and robust pose labeling without compromising privacy. Our approach represents one of the first to harness this complementary relationship, enabling precise, scalable annotation of mmWave point clouds without invoking visual sensing at any stage—effectively resolving the longstanding trade-off between privacy and annotation fidelity that constrains prior mmWave HPE datasets.

## 3. Privacy-First Data Construction

### 3.1. Privacy-by-Design Framework

The core architecture of our data collection framework implements a strict privacy-by-design paradigm through the complete exclusion of optical sensors. Millimeter-wave radar captures motion data exclusively as sparse 3D point clouds, $P=\{p_{i}|p_{i}=\left(x,y,z,v,I\right)\}\in R^{5}$, inherently incapable of reconstructing visual identifiers such as facial features or body morphology. Simultaneously, the VDSuit-Full motion-capture system employs several inertial measurement units (IMUs), directly generating joint coordinates without recording any visual representations. Additionally, the point cloud representation provides information-theoretic anonymity through dimensionality reduction. Crucially, the system operates without supplemental RGB, infrared, or depth cameras that could compromise privacy, establishing a new standard for ethically compliant human sensing.

### 3.2. Action Design

Action sequences in the dataset were carefully curated to address critical gaps in privacy-sensitive monitoring scenarios, particularly those relevant to healthcare, eldercare, and smart home environments. A total of 11 representative human actions were selected, spanning a spectrum from low-dynamic daily tasks to high-risk emergency motions. These actions were classified into three functional categories:

- Basic gestures, such as single-hand waving and shoulder touching, reflect common interactive movements that may be used in gesture-controlled interfaces or passive monitoring of user activity.
- Activities of daily living (ADLs), including sitting-to-standing transitions and simulated dressing motions, are essential for assessing autonomy and physical function—especially in bathroom or bedroom settings, where visual sensors are categorically prohibited due to privacy regulations.
- Critical fall scenarios, including forward, backward, and lateral falls, were deliberately designed and validated by referencing the established fall-detection literature. These sequences simulate the most frequent and injurious fall patterns observed in elderly care environments.

Additional movements, such as controlled leg raises, static balancing poses, and transitional weight shifts, were incorporated to increase diversity in joint kinematics and center-of-mass dynamics. Importantly, to model viewpoint and orientation variance, each action was performed at three different yaw angles relative to the radar’s boresight (0°, 90°, and 180°), ensuring robustness of the dataset against rotation-dependent occlusion and sensor placement biases.

Each sequence spans approximately 5 to 15 s, capturing the complete temporal evolution of the motion from initiation to resolution. This full-trajectory coverage is crucial for downstream learning tasks such as sequence-based pose estimation, activity classification, and time-series fall prediction. The design ensures that mmFree-Pose reflects realistic movement complexity under privacy-preserving conditions, providing a benchmark that is both ethically compliant and technically challenging.

### 3.3. Data Preprocessing Pipeline

**Figure 2:**
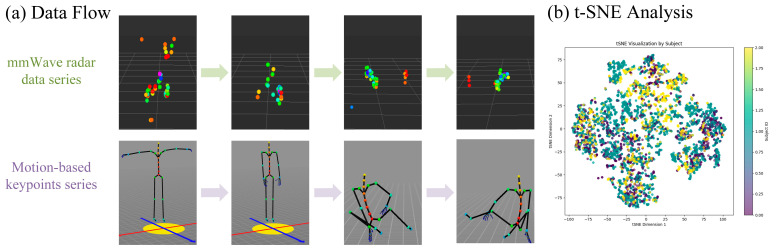
Temporal synchronization and feature Analysis of mmFree-Pose data: (**a**) time-aligned data flow—synchronized mmWave radar point clouds (**top**) and motion-capture keypoints (**bottom**); and (**b**) t-SNE visualization of pose feature embeddings (colored by participant).

Using the IWR6843 ISK radar sensor from Texas Instruments, raw data underwent rigorous preprocessing to enhance quality while preserving privacy attributes. Radar point clouds were filtered to remove non-human points and environmental noise, retaining only clusters corresponding to body segments. Moreover, precise alignment between radar and motion-capture systems was achieved through temporal signal. Additionally, spatial calibration employed a hip-centered coordinate system where $J_{\mathrm{hip}}=\left(0,0,0\right)$, with Kabsch algorithm optimization minimizing residual error between radar centroids and mocap joint positions. Skeletal data was normalized to the hip joint origin through affine transformation, $\hat{J_{k}}=J_{k}-J_{hip}$, ensuring consistent coordinate frames despite participant movement. To compensate for radar sparsity, we applied a simple temporal fusion technique by averaging five consecutive frames to enhance point density. While this approach helps reduce transient noise and improve annotation alignment with IMU data, it may also introduce temporal smoothing effects that can blur rapid motions. In future work, we aim to adopt more advanced temporal denoising methods, such as Kalman filtering or learned temporal smoothers, to better preserve dynamic motion characteristics. Final anonymization scrubbed all metadata, including timestamps, locations, and anthropometric records, assigning de-identified participant codes (P01–P03). The resulting dataset comprises 4534 samples with matched point clouds and 23-joint annotations, partitioned into training (80%), validation (10%), and test sets (10%) while maintaining temporal continuity within sequences. A data exemplar is provided in Figure 2a.

Furthermore, to visually observe the impact of subjects on data distribution and action categories, we performed t-SNE visualization on the data samples, as shown in Figure 2b. The visualization results revealed that the inter-subject variability in point cloud distributions is relatively small compared to the variability introduced by different poses and actions. This suggests that the model’s performance is more influenced by pose diversity than by individual anthropometric differences.

## 4. Data Value Validation

### 4.1. Experimental Protocol

**Figure 3:**
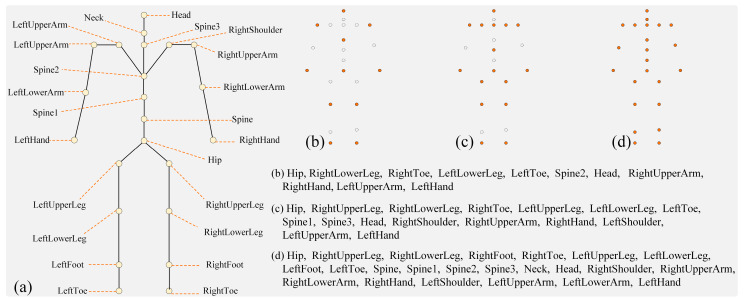
Three different joint settings are used in our experiments to assess the model’s robustness under varying joint counts. (**a**) displays a full human body skeleton with 23 labeled joint points. (**b**–**d**) show point sets of body joints with 11, 16, and 23 joints, respectively. The incremental number of joints allows us to evaluate the model’s performance across different levels of complexity.

The validation framework employed standardized evaluation metrics and baseline models to objectively quantify dataset efficacy. Point Transformer [40], PointNet [41], and DGCNN [42] architectures served as reference models, representing fundamental point cloud processing approaches widely adopted in mmWave HPE research. The models are evaluated under three different settings—11, 16, and 23 joints—representing varying levels of model complexity and granularity in estimating human poses; more details are illustrated in Figure 3.

**Figure 4:**
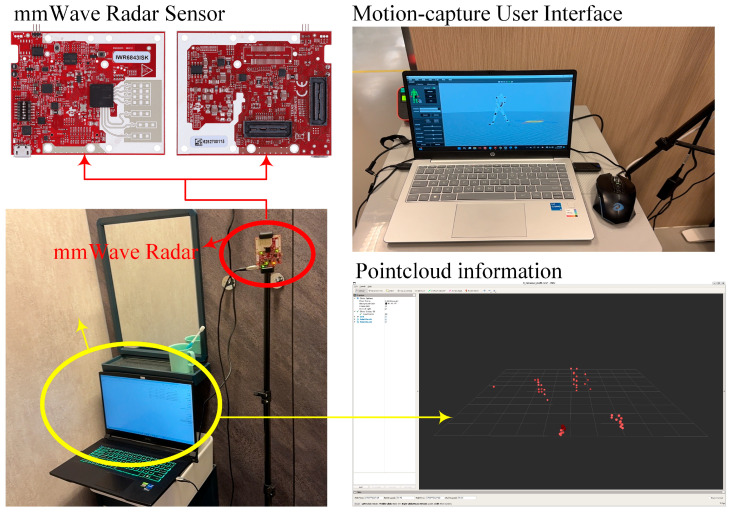
(**Left**) Hardware setup: mmWave radar sensor and laptop terminal for real-time data processing. (**Right**) Motion-capture interface displaying synchronized point cloud visualization.

Performance was assessed through three complementary metrics: Percentage of Correct Keypoints within a 10 cm threshold (PCK@5 cm), Mean per Joint Position Error (MPJPE), and Average Precision across Object Keypoint Similarity thresholds from 0.5 to 0.95 (AP). Statistical significance against existing datasets was established via Wilcoxon signed-rank tests with α = 0.01 confidence level. The experiment setup is illustrated in Figure 4.

### 4.2. Benchmark Performance

**Figure 5:**
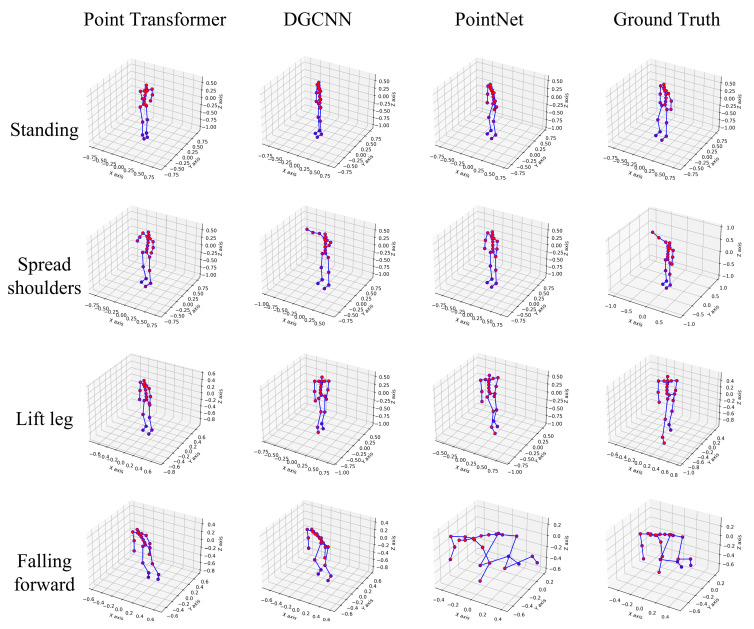
Qualitative comparison of pose estimation models on privacy-sensitive actions.

As shown in Table 1, PointNet demonstrates superior performance across all joint configurations and metrics, achieving State-of-the-Art results on the mmFree-Pose dataset. For the critical 23-joint evaluation, PointNet reaches the lowest Mean Localization Error (12.39 cm vs. DGCNN’s 13.01 cm) while improving PCK (47.18% vs. Point Transformer’s 40.89%) and AP (95% vs. DGCNN’s 89%). Notably, its advantage widens with simpler skeletons (16 joints: 44.17% PCK higher than DGCNN), suggesting exceptional efficiency in core joint tracking. Surprisingly, the attention-based Point Transformer underperforms despite its theoretical capacity, indicating sparse mmWave point clouds (∼50 points/frame) may hinder complex architectural learning. These results establish PointNet as the optimal baseline for privacy-sensitive HPE applications requiring balance between accuracy and computational efficiency. Visualization results, provided in Figure 5, further emphasize the robustness of deep learning approaches in accurately estimating human poses across different keypoint configurations, regardless of complexity.

Error distribution: As detailed in Table 2, joint-wise error analysis reveals a consistent hierarchy in prediction accuracy across all evaluated models. Core joints—notably the hip and spine—demonstrate substantially lower MPJPEs compared to peripheral joints. For example, hip localization errors range from 0.001 cm (DGCNN) to 0.202 cm (Point Transformer), while spine-related joints maintain sub-2 cm accuracy. In contrast, extremities such as hands and feet exhibit much higher error rates, ranging from ~18 cm to over 27 cm, with the left hand reaching 26.27 cm error in Point Transformer and 23.24 cm in PointNet.

This disparity is primarily attributable to point cloud sparsity and radar reflectivity variance: the torso typically generates denser and more stable radar returns due to its larger surface area and proximity to the radar boresight, whereas smaller appendages, like wrists, toes, and fingers, contribute weaker, noisier returns that may intermittently disappear from the radar’s field of view during rapid motion or occlusion. Moreover, limb extremities exhibit greater range of motion, increasing prediction difficulty under non-uniform sampling.

Efficiency trade-offs: The further comparison for real-world deployment feasibility is summarized in Table 3. Computational benchmarks reveal that PointNet achieves an optimal trade-off between efficiency and accuracy, making it well-suited for latency-sensitive and resource-constrained environments such as smart home hubs, mobile health monitors, or embedded fall detection units.

Specifically, PointNet requires only 11.45 million floating-point operations (FLOPs)—largely fewer than DGCNN (54.69 M)—while maintaining a low inference latency of 2 milliseconds per frame. This low computational footprint ensures high responsiveness even on non-GPU devices or low-power processors, which are common in medical wearables or IoT edge systems. While Point Transformer exhibits superior parameter efficiency (298.18 K parameters vs. PointNet’s 349.12 K), its higher FLOPs (24.48 M) indicate more intensive real-time processing demands, which—combined with its significantly lower fall detection PCK—limit its viability in clinical applications, where both accuracy and reaction time are essential.

Collectively, these findings establish PointNet as the most deployable architecture under practical constraints. Its balance of accuracy, low latency, and moderate complexity makes it ideal for real-world HPE systems deployed in privacy-sensitive, real-time, and resource-limited environments.

### 4.3. Performance on Complex Motions

In challenging fall scenarios—one of critical but technically demanding tasks for human pose estimation—PointNet demonstrates superior robustness compared to competing architectures (Table 4). Specifically, it achieves an MLE of 16.73 cm, representing a 16.2% improvement over DGCNN (18.99 cm) and a 24.4% improvement over Point Transformer (22.13 cm). Furthermore, it records the highest AP of 90%, indicating a reliable capacity to localize joints even under rapid, chaotic movement conditions common in falls.

This performance is particularly significant given the inherent difficulties in radar-based estimation of high-velocity limb trajectories. Fall events typically involve sudden, multi-joint rotations and ground contact within a short time span, often accompanied by partial occlusion or transient disappearance of extremity reflections in the radar signal. Such dynamics challenge model inference due to sparse and noisy point clouds, and degrade spatial coherence across frames.

Despite these constraints, PointNet achieves a Peak Correct Keypoint (PCK) of 43.93%, narrowly outperforming DGCNN (43.66%) and clearly surpassing Point Transformer (33.34%). The margin suggests that simpler architectural designs may generalize better to data-sparse, high-variance scenarios than transformer-based networks, which tend to rely on dense attention patterns ill-suited for radar sparsity (typically <200 points/frame). The reliability of PointNet in fall detection extends its potential for real-time applications.

## 5. Reproducibility and Applications

The mmFree-Pose dataset enables transformative applications in privacy-sensitive domains where visual monitoring is ethically or legally prohibited. In clinical settings, its high-fidelity fall sequences support developing non-invasive emergency alert systems for elderly care facilities, operating reliably in darkness without compromising patient dignity. For smart homes, gesture recognition during activities like sitting-to-standing control of bathroom appliances, while low-latency inference (e.g., PointNet at 2 ms) facilitates deployment on resource-constrained edge devices. Indus-trial safety monitoring further benefits from the inherent anonymity of point clouds, enabling worker activity tracking in hazardous zones.

Despite these advances, three limitations warrant consideration. First, peripheral joint localization remains challenged during dynamic motions, with high hand errors (Table 4) due to radar shortcomings—a fundamental constraint of mmWave sparsity. Second, demographic coverage is currently limited to three subjects (age 25–32, BMI 18.5–24.9), restricting generalizability to diverse populations such as pediatric or obese individuals. Third, while the dataset includes multiple orientations, it lacks arbitrary view angles and continuous transitions between poses. This discrete setup limits the ecological realism of natural human behavior, where orientation and pose evolve fluidly. Finally, data collection occurred in controlled lab environments, omitting real-world interference like moving furniture or pets, potentially inflating performance metrics versus field deployments.

Future work will address these constraints through targeted innovations. Demographic diversity will be expanded via recruiting more representative subjects without additional privacy-sensitive captures. Finally, we will initiate real-world validation in assisted living facilities, incorporating environmental clutter to bridge the lab-to-field gap. These efforts will solidify mmFree-Pose as a foundational resource for privacy-centric human sensing, advancing technologies that harmonize safety with uncompromising ethical standards.

## 6. Conclusions

This paper presents mmFree-Pose, the first dedicated mmWave radar dataset explicitly designed to address the critical challenge of privacy preservation in human pose estimation (HPE). By establishing a novel visual-free acquisition framework, we eliminate the ethical and technical risks associated with optical sensors while achieving medical-grade annotation accuracy through synchronized inertial motion capture. The dataset covers 11 safety-critical and socially relevant actions—from basic gestures to high-risk falls—captured under multiple orientations relative to the radar. Each sample provides raw 3D point clouds (with Doppler velocity and intensity) paired with precise 23-joint skeletal annotations, enabling robust model training without compromising subject anonymity.

Our experimental validation demonstrates that mmFree-Pose bridges the gap between RF sensing and privacy-critical applications. Benchmark evaluations reveal that PointNet achieves a State-of-the-Art performance on complex motions, outperforming transformer-based and graph convolutional counterparts. This underscores its suitability for latency-sensitive deployments in eldercare monitoring or smart home systems, where low computational overhead (11.45 M FLOPs, 2 ms inference time) is essential. Crucially, the dataset’s design directly resolves the longstanding trade-off between annotation fidelity and privacy compliance, setting a new standard for ethically grounded human sensing.

Despite its contributions, limitations remain: peripheral joint localization errors persist due to radar sparsity, demographic diversity is currently limited, and real-world environmental interference was not modeled. Future work will expand subject diversity (e.g., age and BMI), integrate multi-radar configurations to mitigate occlusion, and validate the framework in operational settings such as assisted-living facilities. We aim to catalyze advancements in privacy-preserving HPE, ultimately enabling technologies that safeguard dignity while enhancing safety in sensitive environments.

## Figures and Tables

**Table 1:** Quantitative comparison of 3D deep HPE methods on curated datasets. Arrows show optimization directions: ↑ for higher-better (PCK, AP); ↓ for lower-better (MLE).

Approach	11 Joints			16 Joints			23 Joints		
	MLE↓	PCK↑	AP↑	MLE↓	PCK↑	AP↑	MLE↓	PCK↑	AP↑
Point Transformer	16.37	37.93	84	14.22	40.21	90	14.31	40.89	91
PointNet	14.39	42.19	91	10.61	44.17	94	12.39	47.18	95
DGCNN	14.86	38.49	85	13.06	41.13	90	13.01	42.01	89

**Table 2:** Mean per Joint Position Error (MPJPE) is used to measure the average Euclidean distance between the predicted keypoints and the ground truth for each joint, where a lower MPJPE indicates higher accuracy. The comparison of several benchmarks is conducted on the full dataset, using a setting of 23 joints for evaluation.

	Approach	Point Transformer	PointNet	DGCNN
Keypoint				
Hip		0.202	0.093	0.001
Right upper leg		6.088	4.118	4.054
Right lower leg		16.259	14.449	15.854
Right foot		21.383	18.981	20.893
Right toe		24.712	20.836	23.029
Left upper leg		6.403	4.250	4.229
Left lower leg		15.873	14.495	14.296
Left foot		20.323	18.303	17.995
Left toe		23.127	19.719	19.505
Spine		1.954	1.833	1.884
Spine 1		3.966	3.880	3.911
Spine 2		6.384	6.240	6.308
Spine 3		8.891	8.746	8.707
Neck		11.719	11.397	11.275
Head		13.786	13.276	13.061
Right shoulder		10.992	9.912	9.954
Right upper arm		17.584	13.608	13.538
Right lower arm		20.406	15.961	17.114
Right hand		27.221	21.990	25.288
Left shoulder		9.884	9.854	9.832
Left upper arm		16.090	13.235	13.112
Left lower arm		19.670	16.519	18.219
Left hand		26.269	23.236	27.215

**Table 3:** The computational comparison of model components at FLOPs, parameters, and inference time per frame.

Approach	23 Joints		
	FLOPs (M)	Parameter (K)	Inference Time (ms)
Point Transformer	24.48	298.18	2
PointNet	11.45	349.12	2
DGCNN	54.69	594.88	3

**Table 4:** Quantitative comparison of 3D deep HPE methods on curated datasets in complicated fall patterns. Arrows show optimization directions: ↑ for higher-better (PCK, AP); ↓ for lower-better (MLE).

Approach	23 Joints		
	MLE↓	PCK↑	AP↑
Point Transformer	22.13	33.34	60
PointNet	16.73	43.93	90
DGCNN	18.99	43.66	70

## Data Availability

The original contributions presented in this study are included in the article. Further inquiries can be directed to the corresponding authors.
